# Supplementary material for: The Arabic Version of the Impact of Event Scale-Revised: Psychometric Evaluation among Psychiatric Patients and the General Public within the Context of COVID-19 Outbreak and Quarantine as Collective Traumatic Events
Source: J Pers Med. 2022 Apr 24;12(5):681. doi: 10.3390/jpm12050681 (PMC9144426; doi:10.3390/jpm12050681)
Supplement: Supplementary file 1 [file jpm-12-00681-s001.zip › Arabic Version of the Impact of Event Scale-Revised (IES-R).PDF]

Arabiska

IES - R

أذكر ان كنت قد عانيت او عانيتي من احدى او بعض الأعراض التالية خلال الايام السبعة الأخيرة.

١- (١٨٣) أُذكَّر باستمرار رغم ارادتي بالذي حصل

لا على الأطلاق = 0	قليلاً = ١	احياناً = ٢	بعض الأحيان =٣	كثيراً =٤

٢- (١٨٤) احاول ان لا اغضب عندما افكر بالذي تعرضت له، او عندما يذكرني شخص ما بذلك

لا على الأطلاق = 0	قليلاً = ١	احياناً = ٢	بعض الأحيان =٣	كثيراً =٤

٣- (١٨٥) لقد حاولت ان انزعها من ذاكرتي.

لا على الأطلاق = 0	قليلاً = ١	احياناً = ٢	بعض الأحيان =٣	كثيراً =٤

٤- (١٨٦) اجد صعوبة في النوم او انام بقلق

لا على الأطلاق = 0	قليلاً = ١	احياناً = ٢	بعض الأحيان =٣	كثيراً =٤

٥- (١٨٧) اتضايق من احاسيس قوية (عارمه) تعود باستمرار عندما افكر بالحوادث

لا على الأطلاق = 0	قليلاً = ١	احياناً = ٢	بعض الأحيان =٣	كثيراً =٤

٦- (١٨٨) ارى الذي حدث في الحلم

لا على الأطلاق = 0	قليلاً = ١	احياناً = ٢	بعض الأحيان =٣	كثيراً =٤

٧- (١٨٩) احاول تجنب كل مايذكرني بما حدث

لا على الأطلاق = 0	قليلاً = ١	احياناً = ٢	بعض الأحيان =٣	كثيراً =٤

٨- (١٩٠) لدي احساس بان الذي حدث ليس واقعياً وانه في الحقيقة لم يحصل ابداً

لا على الأطلاق = 0	قليلاً = ١	احياناً = ٢	بعض الأحيان =٣	كثيراً =٤

٩- (١٩١) حاولت تجنب التكلم عن ماحدث

لا على الأطلاق = 0	قليلاً = ١	احياناً = ٢	بعض الأحيان =٣	كثيراً =٤

١٠- (١٩٢) صور في الذاكرة عن ماحدث خطرت في ذهني

لا على الأطلاق = 0	قليلاً = ١	احياناً = ٢	بعض الأحيان =٣	كثيراً =٤

Arabiska

١١- (١٩٣) اشياء في محيطي (حولي) تذكرني بالذي حصل

لا على الأطلاق = 0	قليلاً = ١	احياناً = ٢	بعض الأحيان =٣	كثيراً =٤

١٢- (١٩٤) انا ادرك بانه لايزال لدي احاسيس قوية لها علاقة بالذي حدث، وليس باستطاعتي التخلص منها

لا على الأطلاق = 0	قليلاً = ١	احياناً = ٢	بعض الأحيان =٣	كثيراً =٤

١٣- (١٩٥) ابذل جهدي لتجنب التفكير بما حدث

لا على الأطلاق = 0	قليلاً = ١	احياناً = ٢	بعض الأحيان =٣	كثيراً =٤

١٤- (١٩٦) كل مايذكرني بالذي حصل، يوقظ لدي احاسيس قوية

لا على الأطلاق = 0	قليلاً = ١	احياناً = ٢	بعض الأحيان =٣	كثيراً =٤

١٥- (١٩٧) احاسيس بالنسبة لما حدث هي نوعاً ما متزعزعة

لا على الأطلاق = 0	قليلاً = ١	احياناً = ٢	بعض الأحيان =٣	كثيراً =٤

١٦- (١٩٨) شعرت باني غاضب، مغتاظ وسريع الانفعال

لا على الأطلاق = 0	قليلاً = ١	احياناً = ٢	بعض الأحيان =٣	كثيراً =٤

١٧- (١٩٩) شعرت باني مرتعش (مرتجف) وسريع الخوف

لا على الأطلاق = 0	قليلاً = ١	احياناً = ٢	بعض الأحيان =٣	كثيراً =٤

١٨- (٢٠٠) اكتشف باني اتصرف واشعر وكأن الذي حدث سوف يتكرر حدوثه ثانية

لا على الأطلاق = 0	قليلاً = ١	احياناً = ٢	بعض الأحيان =٣	كثيراً =٤

١٩- (٢٠١) لدي صعوبة في النوم

لا على الأطلاق = 0	قليلاً = ١	احياناً = ٢	بعض الأحيان =٣	كثيراً =٤

٢٠- (٢٠٢) لدي صعوبة في التركيز

لا على الأطلاق = 0	قليلاً = ١	احياناً = ٢	بعض الأحيان =٣	كثيراً =٤

٢١- (٢٠٣) عندما أذكّر بالذي حصل، ينتابني اعراض كالتعرق، صعوبة في التنفس، دوخة او خفقان في القلب

لا على الأطلاق = 0	قليلاً = ١	احياناً = ٢	بعض الأحيان =٣	كثيراً =٤

٢٢- (٢٠٤) انا فطن باستمرار

لا على الأطلاق = 0	قليلاً = ١	احياناً = ٢	بعض الأحيان =٣	كثيراً =٤

(٠,١,٣,٥)
