# Supplementary material for: The Arabic Version of the Impact of Event Scale-Revised: Psychometric Evaluation among Psychiatric Patients and the General Public within the Context of COVID-19 Outbreak and Quarantine as Collective Traumatic Events
Source: J Pers Med. 2022 Apr 24;12(5):681. doi: 10.3390/jpm12050681 (PMC9144426; doi:10.3390/jpm12050681)
Supplement: Supplementary file 1 [file jpm-12-00681-s001.zip › Supplementary Table S1.pdf]

Table S1. Factors and corresponding items on every model

Factors	Items comprising factors in each model				
	**Model 1**	**Model 2/Model 3○**	**Model 4**	**Model 5/Model 6○**	**Model 7/Model 8○/ Model 9▲**
COVID-19 trauma	1-22				
Avoidance		5,7,8,11,12,13,17,22	5, 8, 11, 22	8, 11, 22	5,8,11,17,22
Intrusion		1,2,3,6,9,14,16,20	1, 3, 6, 9, 14, 16, 19, 20	1, 3, 5, 6, 9, 14, 16, 19, 20	1,3,6,9,20
Hyperarousal		4,10,15,18,19,21	4, 10, 21	4, 10	16,18,19, 21
Difficulty sleeping/concentrating			2, 15, 18	2, 15, 18	
Numbing			7, 12, 13, 17	7, 12, 13, 17, 21	7,12,13,14
Sleep problems					2,15
Irritability/dysphoria					4,10

○: Bifactor model comprising a general factor in addition to domain-specific factors; ▲: second order factor.
