# Supplementary material for: The Arabic Version of the Impact of Event Scale-Revised: Psychometric Evaluation among Psychiatric Patients and the General Public within the Context of COVID-19 Outbreak and Quarantine as Collective Traumatic Events
Source: J Pers Med. 2022 Apr 24;12(5):681. doi: 10.3390/jpm12050681 (PMC9144426; doi:10.3390/jpm12050681)
Supplement: Supplementary file 1 [file jpm-12-00681-s001.zip › Supplementary Table S2.pdf]

Supplementary Table S2. Goodness-of-fit indices for structural equation models examining interactions among factors comprising the Arabic version of the Impact of Event Scale-Revised (IES-R)

**Sample**	χ^2	*df*	**p**	**CFI**	**TLI**	**RMSEA**	**RMSEA 90% CI**	**SRMR**
Quarantine	6.334	6	0.387	0.999	0.998	0.016	0.000 to 0.092	0.0221
Sample 1	5.663	5	0.340	0.999	0.996	0.028	0.000 to 0.114	0.0226
Sample 2	5.902	3	0.116	0.999	0.995	0.031	0.000 to 0.069	0.0100

χ^2: chi-square; df: degrees of freedom; CFI: comparative fit index; TLI: Tucker–Lewis index; RMSEA: root mean square error of approximation; CI: confidence interval; SRMR: standardized root mean residual.
